# Supplementary material for: The effect of distant connections on node anonymity in complex networks
Source: Sci Rep. 2024 Jan 12;14:1156. doi: 10.1038/s41598-023-50617-z (PMC10784523; doi:10.1038/s41598-023-50617-z)
Supplement: Supplementary file 1 — Supplementary Information. [file 41598_2023_50617_MOESM1_ESM.pdf]

Supplementary information

d-k-Anonymity for $d = 3$, $d = 4$ and $d = 5$

Supplementary Figure 1 shows additional results for Section "Beyond the ego network in graph models" of the main manuscript. The figure is similar to Fig. 2, showing the fraction of unique nodes in graph models with knowledge of the d-neighborhood, for $d = 3$, $d = 4$ and $d = 5$. The figures show that further increasing the distance beyond a value 2 has a minimal de-anonymizing effect for these graph models; the largest effect is observed when moving from distance 1 to distance 2, as shown in Fig. 2.

Supplementary Figure 2 shows additional results for Section "Beyond the ego network in real-world network data" of the main manuscript, including uniqueness for d-k-anonymity with $d = 1$ up to $d = 5$. The figure is similar to Fig. 5, and includes results shown in Fig. 3 for $k = 1$. The figure shows that information beyond $d = 2$ has only a small to no de-anonymizing effect on these networks.

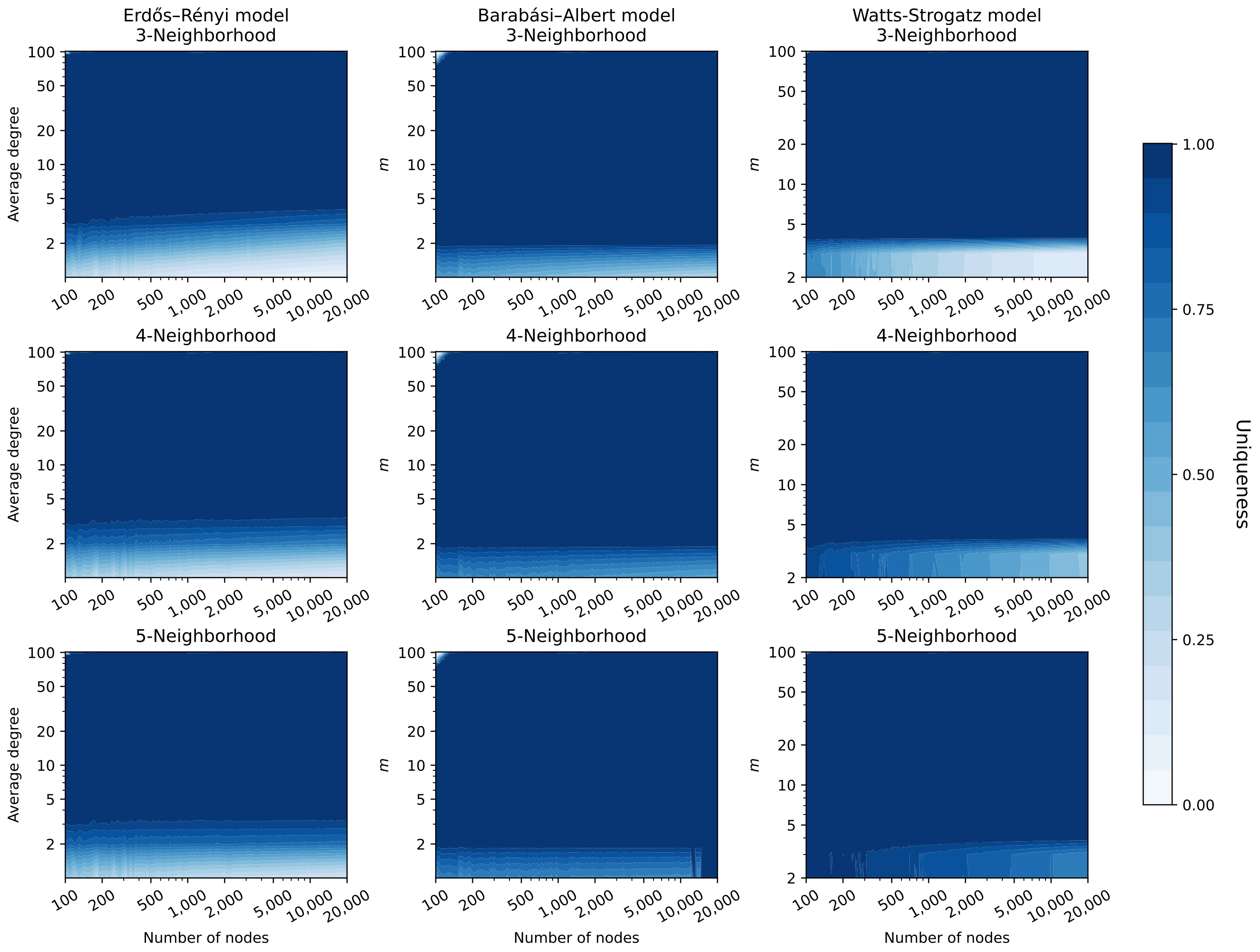

Supplementary Figure 1. Uniqueness maps using d-k-anonymity. Maps show network uniqueness, indicated by color, when using information of the 3-neighborhood (top row), 4-neighborhood (middle row) and 5-neighborhood. Results are shown for the Erdős–Rényi (left), Barabási–Albert (middle) and Watts–Strogatz (right) model with different sizes (horizontal axis) and average degree or m, an equivalent thereof (vertical axis).

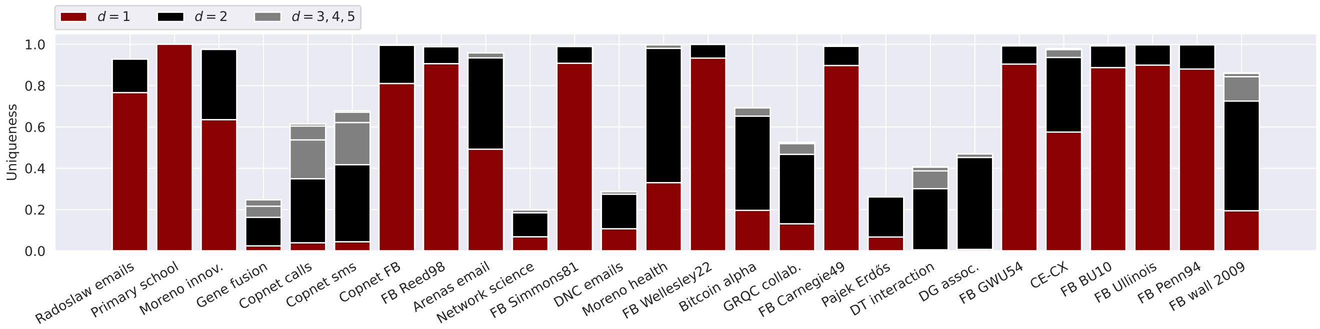

Supplementary Figure 2. Uniqueness in real-world networks for d-k-Anonymity with $d = 1$ up to $d = 5$. Fraction of unique nodes (vertical axis) on different datasets (horizontal axis) when accounting for different levels of information: 1-neighborhood (red), 2-neighborhood (black), 3- 4- and 5-neighborhood (grey).

d-k-Anonymity in dense graph models

Similarly to the uniqueness maps in Fig. 2 and 4 of the main manuscript, we show the uniqueness of graph models with a density up to their maximum degree in Supplementary Fig. 3. A step size of 0.01 is used, and all results are averaged over 10 runs.

Overall, we see that for most of the graph models, for higher densities all nodes become unique. We observe non-uniqueness for graphs with either low densities, or for very high densities. The graphs with uniqueness at high densities are (near)-complete graphs, and can only be clearly distinguished in the top left of these figures for small graphs.

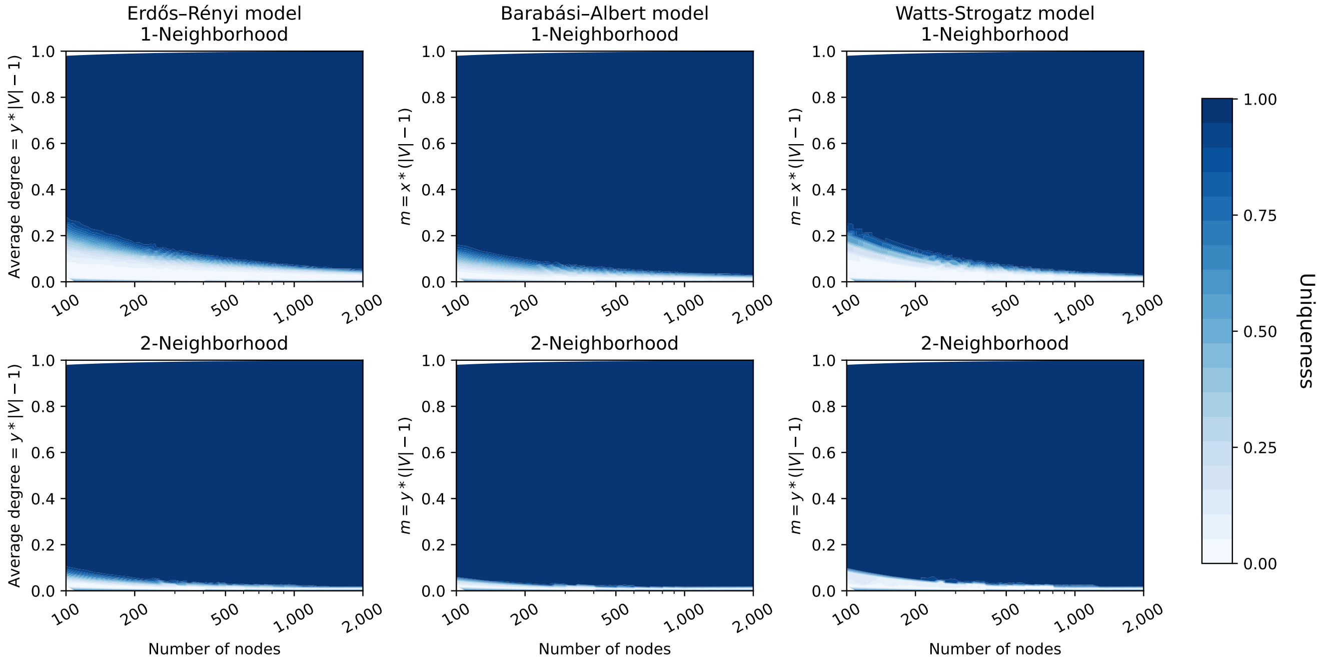

Supplementary Figure 3. Uniqueness maps using d-k-anonymity in dense graph models. Maps show network uniqueness, indicated by color, when using information of the 1-neighborhood (top row) and 2-neighborhood (bottom row). Results are shown for the Erdős–Rényi (left), Barabási–Albert (middle) and Watts–Strogatz (right) model with different sizes (horizontal axis) and average degree or m, an equivalent thereof (vertical axis).

Real-world networks vs. graph models

To compare the results on real-world networks with those obtained for the graph models, Supplementary Fig. 4 shows for each network the position in the uniqueness map, similar to Figures 2 and 4 of the main manuscript, and indicates the uniqueness by color. Similar to the results obtained by Romanini *et al.*[1] and for the graph models, the figure shows that average degree has a large effect on the uniqueness obtained. The number of nodes has an overall smaller effect.

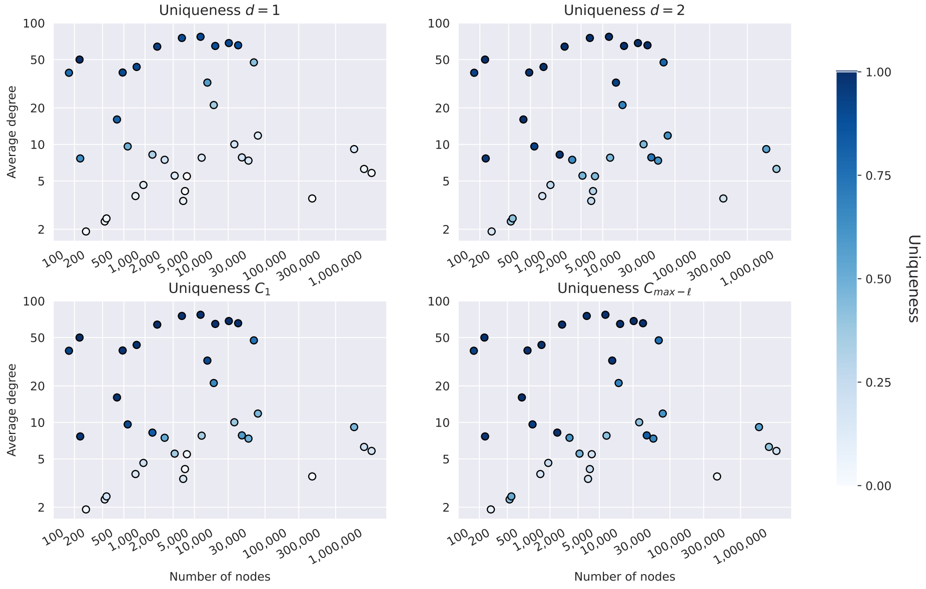

Supplementary Figure 4. Real-world networks positioned in uniqueness maps. For all networks listed in Table 1, their position in the uniqueness map, which is used in Figures 2 and 4 of the main manuscript, is shown and the uniqueness is indicated based on the color.

Graph properties vs. experimental results

To get a better understanding of the obtained results in real-world networks presented in the "Beyond the ego network" and "Anonymity-cascade" sections of the main manuscript, we aim to find which graph properties relate to various uniqueness results and experimental outcomes. The results considered are the uniqueness using 1-k-anonymity, the highest achieved cascading depth $max - \ell$, and the difference obtained in uniqueness by instead of 1-k-anonymity considering 2-k-anonymity, or one level of cascading C_1. Since we have a diverse set of networks from different categories, we compute the Pearson correlation and the corresponding p-value in Supplementary Table 1 for each combination of graph property (computed using igraph[2]) and outcome. Results are computed for the networks in Table 1 of the main manuscript, excluding the four largest networks due to large runtimes for computing several of the properties.

	Uniqueness $d = 1$		$max - \ell$		Uniqueness $(d = 2) - (d = 1)$		Uniqueness $(C_1) - (d = 1)$	
Network property	Pearson correlation	p-value	Pearson correlation	p-value	Pearson correlation	p-value	Pearson correlation	p-value
Nodes	-0.113	5.38E-01	0.203	2.65E-01	-0.123	5.03E-01	0.385	**2.97E-02**
Edges	0.305	8.91E-02	-0.146	4.24E-01	-0.098	5.93E-01	0.063	7.33E-01
Average degree	**0.865**	**1.75E-10**	**-0.574**	**5.85E-04**	-0.068	7.13E-01	-0.322	7.24E-02
Median degree	**0.895**	**4.79E-12**	**-0.621**	**1.48E-04**	-0.041	8.24E-01	**-0.418**	**1.71E-02**
Max degree	**0.599**	**2.89E-04**	-0.344	5.37E-02	-0.077	6.75E-01	-0.266	1.41E-01
Density	**0.412**	**1.93E-02**	**-0.480**	**5.42E-03**	-0.002	9.92E-01	-0.228	2.10E-01
Transitivity	0.007	9.70E-01	-0.263	1.46E-01	-0.001	9.96E-01	-0.035	8.48E-01
Assortativity	0.085	6.44E-01	0.122	5.06E-01	0.122	5.06E-01	0.228	2.09E-01
Diameter	**-0.599**	**2.92E-04**	**0.809**	**2.06E-08**	-0.136	4.58E-01	0.179	3.28E-01
Average path length	**-0.740**	**1.28E-06**	**0.854**	**4.94E-10**	-0.114	5.35E-01	0.112	5.41E-01

Supplementary Table 1. Graph properties and correlation with results. Given various graph measures, denoted in the leftmost column, the Pearson correlation is shown with the outcomes (second to last columns). For each outcome both the Pearson correlation and p-value are given. Values with $p < 0.05$ and Pearson correlation larger than 0.4, or smaller than -0.4, are shown in bold.

The results in Supplementary Table 1 show that there are correlations between various graph properties and both the uniqueness at $d = 1$ and $max - \ell$. For uniqueness, the average, median and maximum degree and density are all positively correlated. Overall, these properties are very related to (or to some extent equivalent to) density, which corresponds to the results obtained by Romanini *et al.*:[1] networks with a higher density overall have a higher uniqueness. This relation can also be seen for graph models in the uniqueness shown in Fig. 2 of the main manuscript. The table also shows that diameter and average path length are negatively correlated to the uniqueness. For $max - \ell$, the reverse holds. This is negatively correlated with density, and positively with diameter and average path length.

For the difference in uniqueness when looking beyond the neighborhood, we only found two substantial correlations when using one level of cascading. Overall a higher maximum degree is is negatively correlated with a larger effect of one level of cascading. For all combinations with a substantial correlation we include a plot with regression line in Supplementary Fig. 5.

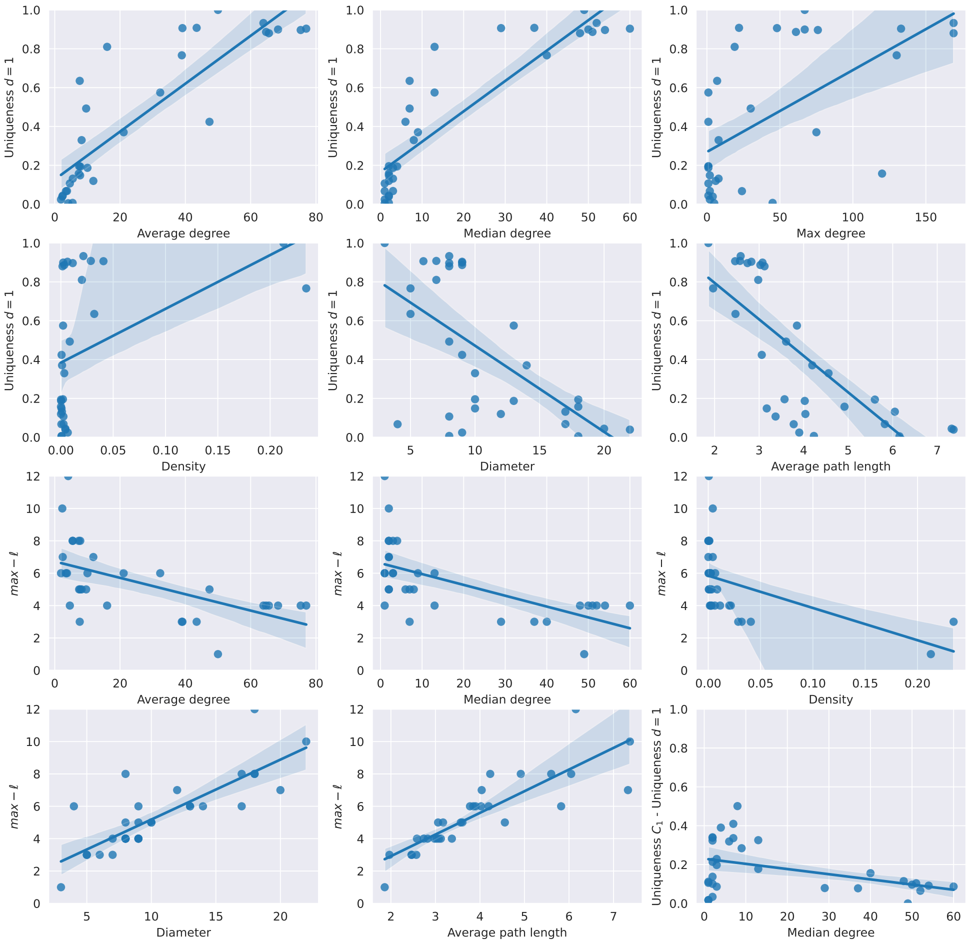

Supplementary Figure 5. Graph properties and outcomes. Each vertical axis denotes a particular experimental outcome (listed in the first row of Supplementary Table 1), each horizontal axis a graph property (listed in the first column of Supplementary Table 1). Only combinations with $p < 0.05$ and Pearson correlation larger than 0.4, or smaller than -0.4 are included.

Effect at different levels of anonymity-cascade

The following figures are supplementary to Section "Anonymity-cascade" of the main manuscript and give additional insights into the effect on anonymity at different levels of *anonymity-cascade*. Supplementary Figure 6 shows the largest level of cascading for the graph models. For these results, *anonymity-cascade* is used for each graph model, the highest level ($max - \ell$) is measured and the average over 10 runs is reported. This overall shows that ($max - \ell$) increases as the graph size increases. In some more sparse graphs with average degree or m around 2 (ER, BA) or 5 (WS), these levels achieve the highest values.

Supplementary Figure 7 shows how many nodes are uniquely identified at each level of cascading in the real-world networks. Overall, the results show that the largest de-anonymizing effect occurs at lower levels of cascading. The cascading step can continue for many levels, but with a much smaller de-anonymizing effect.

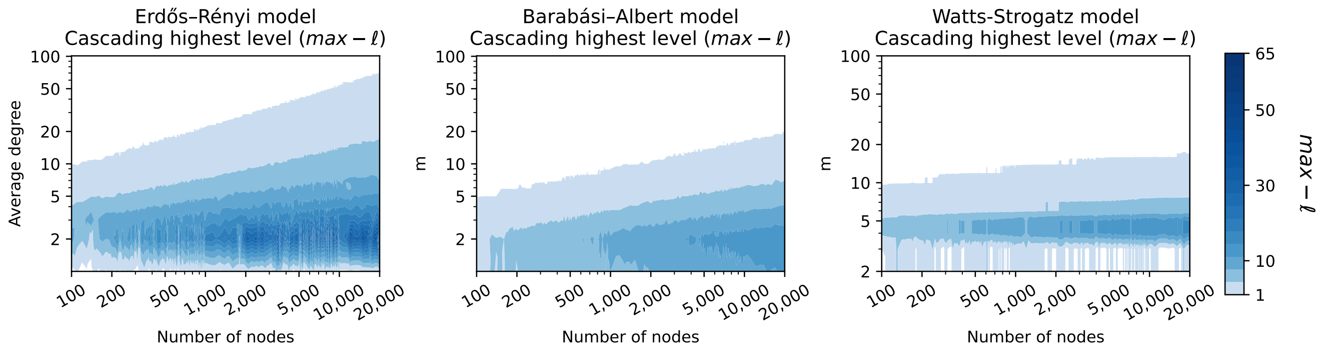

Supplementary Figure 6. Cascading levels in graph models. Results for graph models showing the highest level achieved ($max - \ell$) by the cascading algorithm for ER (left) BA (middle) and WS (right) graphs. All results are averaged over 10 generated graphs.

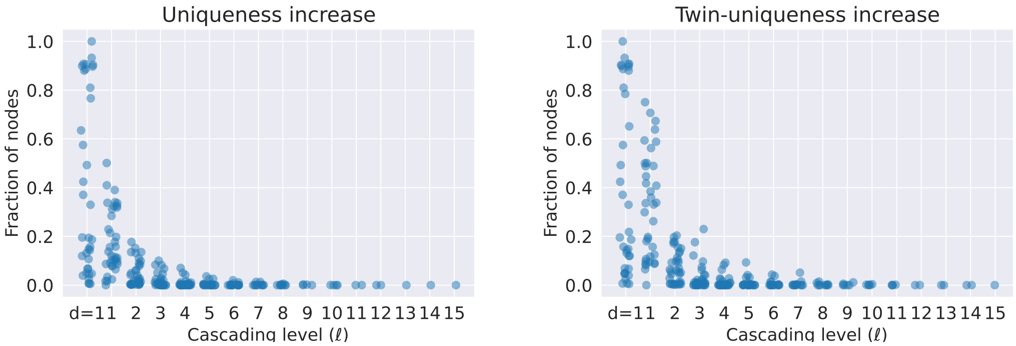

Supplementary Figure 7. Cascading effect per level. Fraction of identified nodes (vertical axis) at $d = 1$ and each subsequent level of cascading when accounting for uniqueness (left) and twin-uniqueness (right). Results summarized over all networks in Table 1 of the main manuscript.

Twin node effect

Supplementary Figure 8 shows the effect of twin nodes and how it influences twin-uniqueness vs. uniqueness. When a network contains a higher fraction of twin nodes, the overall effect is larger.

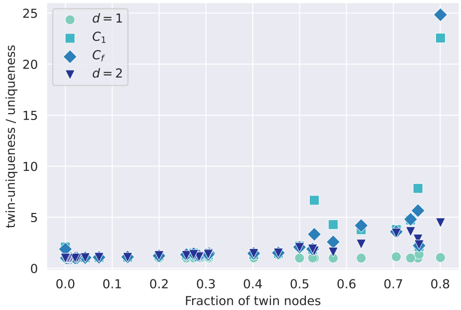

Supplementary Figure 8. Effect of twin nodes on twin-uniqueness. Relation between the fraction of twin nodes in the network (horizontal axis) and how much the uniqueness increases (vertical axis) compared to when twin nodes are not taken into account.

References

1. Romanini, D., Lehmann, S. & Kivelä, M. Privacy and uniqueness of neighborhoods in social networks. *Sci. Reports 11: 20104* (2021).
2. Csardi, G., Nepusz, T. *et al.* The igraph software package for complex network research. *InterJournal, complex systems* **1695**, 1–9 (2006).
